# Dietary resistant starch alleviates *Escherichia coli*-induced bone loss in meat ducks by promoting short-chain fatty acid production and inhibiting Malt1/NF-κB inflammasome activation

**DOI:** 10.1186/s40104-022-00739-7

**Published:** 2022-08-05

**Authors:** Huaiyong Zhang, Simeng Qin, Xiangli Zhang, Pengfei Du, Yao Zhu, Yanqun Huang, Joris Michiels, Quifeng Zeng, Wen Chen

**Affiliations:** 1 College of Animal Science and Technology, Key Laboratory of Animal Biochemistry and Nutrition, Ministry of Agriculture, Henan Agricultural University, Zhengzhou, 450046 China; 2 Laboratory for Animal Nutrition and Animal Product Quality, Department of Animal Sciences and Aquatic Ecology, Ghent University, 9000 Ghent, Belgium; 3 Institute of Animal Nutrition, Key Laboratory for Animal Disease-Resistance Nutrition of China, Ministry of Education, Sichuan Agricultural University, Chengdu, 611130 China

**Keywords:** Bone loss, Malt1/NF-κB signalling, Microbiota, Resistant starch, SCFAs

## Abstract

**Background:**

*Escherichia coli* (*E. coli*) infection in humans and animals usually comes with gut dysbiosis, which is potential culprit to skeletal health, it is still unclear to whether diet interfered gut microbiome changes can be a protective strategy to bone loss development. Here, the effects of resistant starch from raw potato starch (RPS), a type of prebiotic, on *E. coli*-induced bone loss and gut microbial composition in meat ducks were evaluated.

**Results:**

The results showed that dietary 12% RPS treatment improved bone quality, depressed bone resorption, and attenuated the pro-inflammatory reaction in both ileum and bone marrow. Meanwhile, the 12% RPS diet also increased the abundance of Firmicutes in *E. coli*-treated birds, along with higher production of short-chain fatty acids (SCFAs) especially propionate and butyrate. Whereas addition of β-acid, an inhibitor of bacterial SCFAs production, to the drinking water of ducks fed 12% RPS diet significantly decreased SCFAs level in cecum content and eliminated RPS-induced tibial mass improvement. Further, treatment with MI-2 to abrogate mucosa-associated lymphoid tissue lymphoma translocation protein 1 (Malt1) activity replicated the protective role of dietary 12% RPS in *E. coli*-induced bone loss including reduced the inhibition on nuclear factor κB (NF-κB) inflammasome activation, decreased bone resorption, and improved bone quality, which were correlated with comparable and higher regulatory T cells (Treg) frequency in MI-2 and 12% RPS group, respectively.

**Conclusions:**

These findings suggested that the diet with 12% RPS could alleviate *E. coli*-induced bone loss in meat ducks by changing the gut microbial composition and promoting concomitant SCFAs production, and consequently inhibiting Malt1/NF-κB inflammasome activation and Treg cells expansion.

**Graphical Abstract:**

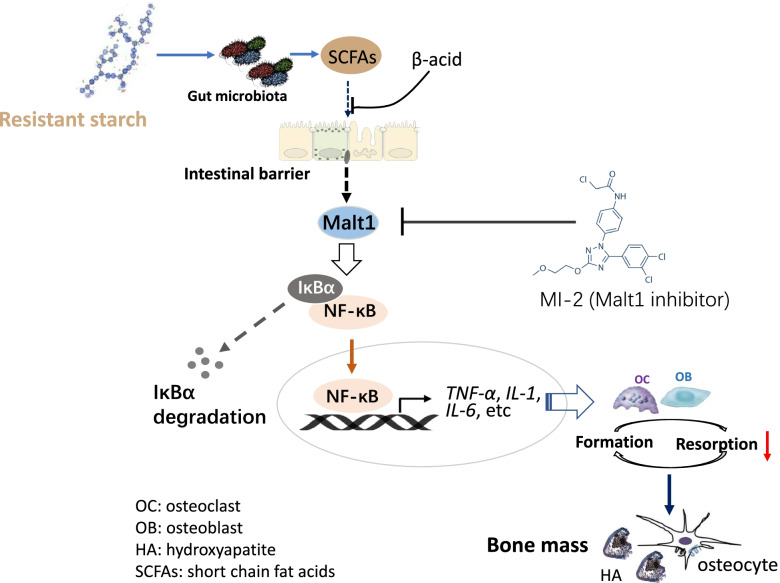

**Supplementary Information:**

The online version contains supplementary material available at 10.1186/s40104-022-00739-7.

## Introduction

Poultry colibacillosis is caused by the Gram-negative bacterium *Escherichia coli* (*E. coli*) that is present in the gastrointestinal tract of humans and animals. As one of potential vehicles for transmission of antimicrobial resistance bacteria, infection by *E. coli* in humans might be as a result of the consumption of poultry meats and eggs. Even worse, resistance to antimicrobials in clinical and veterinary medicine has been increasingly reported in *E. coli* and this has become a public health concern worldwide [1]. In addition to compromised health and performance due to impaired intestinal barrier, disturbed immune function and microbiome profile [2–4], birds infected with pathogenic strains of *E. coli* were also probably associated with bone abnormalities. For example, broilers challenged with *E. coli* involve in vertebral osteomyelitis and arthritis dependent of the diversity of *E. coli* strains [5]. Concerned with meat ducks, both triggered inflammatory response [6] and altered gut microbiome profile [4] by *E. coli* could be potential attributors to bone problems of ducks. Our recent research on broilers indicated that impairment of gut integrity and dysbiosis could induce systemic inflammation to stimulate bone resorption, a process by which the bones were absorbed and broken down by some important enzymes such as V-ATPases and cathepsin K thus reducing bone mass and mechanical properties, which was called “gut-bone” axis [7]. There is also strong evidence that intestinal microbiota could modulate bone mass, depending on immune reaction and their metabolites including short-chain fatty acids (SCFAs) [8]. In addition to acidifying intestinal environment to promoting minerals abruption, including calcium (Ca) and prosperous (P), the protective effects of SCFAs on bone mass were also proved to associate with inhibition of osteoclast differentiation and bone resorption in vitro and in vivo [9]. By comparing germ-free mice with conventionally raised mice it was shown that the presence of microbiota led to lower trabecular and cortical bone mass [10]. A further alteration in gut microbiota via oral antibiotics impaired bone density in mice [11]. Meanwhile, germ-free mice were showing a reduced number of osteoclasts and lower level of interleukin (IL)-6, tumour necrosis factor alpha (TNF-α), and CD4^+^T cells in bone [12, 13], and which were normalized by colonization with gut microbiota from conventionally raised mice [13]. These data suggest that the immune system activated by disturbed microbiota may play a significant role in the loss of bone health.

It is well established that mucosa-associated lymphoid tissue lymphoma translocation protein 1 (Malt1) is an intracellular signalling protein in innate and adaptive immunity [14]. In addition to acting as a scaffold to active the nuclear factor-κB (NF-κB) signalling, a key regulator in the secretion of inflammatory cytokines, Malt1 proteolytic activity also fine-tunes gene expression in myeloid cells and nonimmune cells [14]. More specifically, Malt1 paracaspase activity enhanced NF-κB activation by cleaving substrates such as A20 and RelB. Of note, these substrates have previously been associated with osteoclastogenesis, i.e., A20 deficiency of myeloid cells resulted in increased osteoclastogenesis in mice [15]. RelB-mediated noncanonical NF-κB activation is required for full receptor activator for nuclear factor-κB ligand (RANKL)-induced osteoclast maturation [16]. Furthermore, RANKL signalling also promoted the activation of different transcription factors including NF-κB [17] and nuclear factor of activated T cells cytoplasmic 1 (NFATc1), the latter is a master regulator of osteoclast differentiation [18]. Treatment with MI-2, a Malt1 inhibitor, dose-dependently attenuated symptoms of colitis in mice via inhibiting NF-κB inflammasome activation [19]. Inhibition of the proteolytic activity of Malt1 with Mepazine in mouse bone marrow precursor cells (BMCs) was strongly related with the inhibition of RANKL-induced formation of osteoclasts, as well as the expression of several osteoclast markers, such as tartrate-resistant acid phosphatase (TRAP), cathepsin K, and calcitonin [20]. It thus implied that Malt1 might be the intermediary key factor to translate gut dysbiosis and concomitant inflammatory response into reduced bone health.

Because of the susceptibility of meat duck to *E. coli* and the resistance problem, the poultry industry is seeking alternatives that help to minimize the indiscriminate use of antibiotics and improve animal health and welfare. Among the alternatives, resistant starch (RS) has been particularly attractive since RS could be consumed by microbiota anchored hindgut in a mutualistic relationship with the host, which are associated with intestinal health [21, 22]. Ongoing research has provided important evidence for the health benefits of RS on improving biochemical functions in the intestinal tract in human [22] and pigs [23]. Data from our recent research on meat ducks showed that dietary supplement of 12% raw potato starch (RPS) as the resource of type II RS can thicken the mucosal layer, tighten the gut barrier, and attenuated inflammatory markers [24, 25], accompanying with the alteration in the composition of the intestinal microbiota, including the increases in *Bifidobacterium*, *Lactobacillus*, and *Bacteroides* [24]. Therefore, it is justifiable to speculate dietary RS could be used as a mediator of improved bone health in meat ducks through its actions on the “gut-bone” axis, which was supported by the capacity of RS to attenuate the bone loss in ovariectomized mice through regulating gut microbiota and bone-marrow inflammation [26–28]. One of potential mechanisms underlying the role of RS in “gut-bone” axis is the production of SCFAs, especially propionate and butyrate. They have well established ability to support gut epithelial integrity and produce anti-inflammatory and immunoregulatory functions [29]. As a result, RS was noticed to alleviate collagen-induced arthritis in mice by modulating gut microbiota and promoting concomitant propionate production [30]. Moreover, addition of β-acids or exogenous propionate to the drinking water of mice fed RS eliminated or restored the beneficial effects of RS on collagen-induced arthritis, respectively [30]. It shed new light onto alleviating bone diseases that the complications of *E. coli* infection. Based on this research, we proposed the hypothesis that dietary 12% RPS supplementation is able to reverse bone loss induced by *E. coli* challenge in meat-type ducks via the anti-inflammatory and anti-bone resorptive property of its fermentation products. With this aim, the effects of dietary RS supplementation from RPS on the tibia quality and bone turnover of meat ducks through “gut-bone” axis was investigated in the present study. Additionally, the key molecular factors and potential mechanism that determined how RS alleviates the progression in tibia abnormalities also was explored.

## Materials and methods

### Chemicals

Raw potato starch (RPS; AVEBE Ltd, Veendam, Holland), a typical Type II RS, used in this study and contains 54.72% RS content (dry matter basis) according to our previous analysis [24]. The inhibitor of bacterial SCFAs production β acids extracted from the hops plant (S. S. Steiner Inc. New York, USA). MI-2 (Eternity Bioscience Inc. NJ, USA) was dissolved at a concentration of 30 mmol/L as a stock solution, stored at -20 °C.

### Animals and management

Day-old male Cherry valley ducklings with an initial average body weight of 48 ± 1.34 g were used in this study. All birds were housed in individual cages (0.16 m^2^/bird) in a temperature- and humidity-controlled room. Temperature was maintained at 32 ± 1 °C for the first week and then the temperature was decreased by 2.75 °C at d 7 and 14. The light schedule was 18 L:6 D throughout the experimental period. All diets were isonitrogenous and isocaloric (Additional file 1: Table S1) and formulated in line with National Research Council guidelines [31]. The apparent metabolizable energy of RS used in this formula came from our previous the analysed value [25]. RS in RPS and diets was analysed using an RS assay kit (K-RSTAR; Megazyme Ltd, Wicklow, Ireland), and confirmed proper preparation of experimental diets. At d 7 and 14, after 6 h of fasting, all ducks were weighed, and feed intake was measured on a per cage basis.

### Experimental design

Ducklings were randomly allocated to 3 treatments: non-challenged (Ctrl, fed with basal diet), *E. coli*-challenged (*E. coli*, fed with basal diet), and *E. coli*-challenged with RS (*E. coli*-RS; fed a 12% RPS-supplemented diet and challenged with *E. coli*). On d 7, except for the Ctrl group, which received 0.6 mmol/L of sterilised Luria–Bertani culture, all birds were orally administrated with 0.6 mmol/L of Luria–Bertani culture containing 6 × 10^8^ CFU/mL of *E. coli* O88 twice, 8 h apart, as previous description [3]. In addition, to evaluate the role of SCFAs in bone metabolism, ducklings were randomly assigned to *E. coli*, *E. coli*-RS, and *E. coli*-RS with β acids (*E. coli*-RS + β acids). β acids were added into drinking water at a final concentration of 20 mg/L from 1 d. *E. coli* and *E. coli*-RS birds received pH and sodium matched water. To further confirm the potential mechanism of RS interacted inflammatory response to restore a reduction in *E. coli*-induced bone loss, ducklings were randomly allocated to *E. coli*, *E. coli*-RS, and *E. coli*-MI-2. From d 1 to 14, Both birds of *E. coli* and *E. coli*-RS were injected intraperitoneally phosphate buffer saline, where ducks from *E. coli*-MI-2 were injected intraperitoneally 30 mg/kg MI-2, respectively. Each treatment with 6 replications of 10 birds per replicate.

### Sample and data collection

At d 14, 1 duck of average body weight from each cage was bled via jugular vein after fasting 6 h and centrifuged at 4000 × *g* for 15 min at 4 °C to obtain serum. Subsequently, birds were sacrificed, the ileal content of each bird was gently removed, and the pH value of the content was directly measured using a pH-STAR (Matthuas Inc. Berlin, Germany). Then, the weight and length of ileum were quickly determined. Mid-ileal mucosa, cecal contents, left tibia (the proximal end), and bone marrow were collected and stored (-80 °C) until analysis. Right tibia (the proximal end) was dissected and rapidly immersed in phosphate-buffered formaldehyde for histology analysis. Another 6 ducks (1 bird per cage) were randomly selected and euthanized. The left tibia was removed for micro computed tomography (Micro-CT) analysis. The right tibia was harvested, length, and width (at 50% of length) of tibia were measured after removal of soft tissues.

### In vivo intestinal permeability

For whole intestinal permeability, 15-day-old ducks (one per cage) were received orally fluorescein isothiocyanate dextran (FITC-d, 4.16 mg/kg body weight) 2 h prior to the time of blood collection. Serum fluorescence was analysed using a Gemini XPS Microplate Reader (Molecular Devices, LLC. Sunnyvale, CA) at an excitation/emission wavelength of 485/530 nm. The content of FITC-d transfer into the serum was calculated from standard curves generated by the serial dilution of FITC-d.

### Sequencing of cecal microbiota

According to our recently description [7], the total DNA in cecal content was extracted using a DNA stool mini kit (Qiagen, Valencia, CA, United States). After assessing the integrity and size of DNA, the hypervariable V3-V4 regions of the 16S rDNA gene was amplified. Then, the resulting PCR products were sequenced on an Illumina PE250 platform (BGI, Shenzhen, China). The obtained sequences were processed using FLASH (v1.2.11) and USEARCH (v7.0.1090) for alignment and clustering. All effective reads were clustered into operational taxonomic units (OTUs) with a similarity threshold of 97%. The representative sequence of each OTU was aligned against the Greengene database for taxonomy analysis. As for data analysis, principal coordinate analysis (PCoA) was performed based on the Bray–Curtis dissimilarity calculated by QIIME software and displayed using R software.

### Cecal SCFAs analysis

Approximately 0.5 g of cecal content was diluted with 2 mL of ultrapure water mixed with a uniform, followed by depositing for 30 min and centrifuging at 3000 × *g* for 15 min. 1 mL supernatants were mixed with 0.2 mL ice-cold 25% (w/v) metaphosphoric acid solution and incubated at 4 °C for 30 min. After centrifuging at 11,000 × *g* for 10 min, the SCFAs contents including acetate, propionate, and butyrate were separated and determined by gas chromatograph (Varian CP-3800, USA), as previously described [24].

### Detection of skeletal strength, fat-free weight, and ash

Mechanical strength was performed by the 3-point bending method using the texture analyser (TA. XT Plus; Stable Microsystems) with a constant 50 kg load cell. Loading proceeded at a constant rate (5 mm/min) until a fracture occurred. The load–displacement curve was recorded, and the maximum load of the tibia was directly read from the peak value. Hereafter, fat-free weigh was determined through air-drying for 24 h at room temperature, extracting by ethyl ether for 48 h, and oven dried at 108 °C for 24 h. Subsequently, dry-defatted tibia was ashed in a muffle furnace at 550 °C for 24 h and the ash was measured based on the percentage of dry-defatted weight.

### Micro-CT

Micro-CT imaging was performed using a GE Explore Locus Micro-CT (GE Healthcare, Piscataway, NJ, USA) with instrument settings optimized for calcified tissue visualization at 90 kV. The analysis of the trabecular bone in the proximal end of the tibia (metaphysis) was performed starting from 9 mm below the surface of the condyles and extending 4 mm distally. To exclude denser cortical regions at the bone surface, the outer 0.5 mm of the bone surface was removed from the region of interest. Bone volume/total volume (BV/TV) and thickness (Tb.Th) of trabecular bone were calculated. The average thickness of the structures was measured using the thickness plugin from Bone J as our previous method following our recent methods [7].

### Histological analysis

The proximal end of tibia was fixed in 10% formaldehyde solution for 24 h and decalcified in ethylene diamine tetraacetic acid (Sigma, USA). Tissues were embedded in paraffin and 5 μm sections were stained with tartrate resistant acid phosphatase (TRAP) bone staining using assay kit (Sigma-Aldrich, USA). Histopathological images were collected using a microscope with image analysis software (Nikon Corporation, Tokyo, Japan). The number of osteoclast (N.Oc/BS) was quantified based on the TRAP staining.

### Flow cytometry

Splenocytes were harvested from the spleen. Erythrocytes were lysed with ammonium-chloride-potassium lysing buffer and spleen cells were stimulated for 5 h in RPMI 1640 medium containing 20 ng/mL phorbol myristate acetate, 1 μg/mL ionomycin, and 10 μg/mL brefeldin A (Sigma-Aldrich, St Louis, MO, USA). For T regulatory (Treg) cells were successively stained with anti-CD3, anti-CD4, anti-CD25, and anti-Foxp3 antibodies. The stained cells were rinsed, resuspended, and analysed by BD AccuriC6 flow cytometer with analysis software (BD Biosciences, San Jose, CA, USA).

### Serum biochemistry

Cytokines of IL-1β, IL-10, IL-17, TNF-α concentrations were measured using enzyme-linked immunosorbent assay (ELISA, Meimian Biotechnology Co., Ltd, Jiangsu, China). Ca and P level were measured with Biochemistry Analyzer (Yellow Springs Instrument Co. Inc., Yellow Springs, OH). Serum bone turnover markers including procollagen type I N-terminal propeptide (P1NP) and C-terminal cross-linked telopeptide of type I collagen (CTx), alkaline phosphatase (ALP) and TRAP activity were assayed by ELISA assay (Nanjing Jiancheng Bioengineering Institute, Nanjing, China) following the manufacturers. All samples were tested in triplicate within each assay.

### Gene expression assays

Ileal, tibia, and bone marrow were pulverized, and RNA was extracted using Trizol (Invitrogen Life Technologies, Carlsbad, CA, USA) following the manufacturer’s instructions. Reverse transcription into cDNA and quantitative real-time PCR were performed was performed on ABI 7900HT detection system (Applied Biosystems, CA, USA). The primer sequences for the target genes designed using Primer 3 (Additional file 1: Table S2). Relative gene expression was quantified by normalizing to the expression of glyceraldehyde-3-phosphate dehydrogenase (*GAPDH*) and β-actin.

### Western blotting

Bone marrow (approximately 0.3 g) was ground in liquid nitrogen and lysed using 3 mL of lysis buffer. After centrifugation, the protein content of the supernatant was determined by using bicinchoninic acid protein assay kits (Thermo Fisher Scientific Inc.). The protein lysates were separated by 10% SDS-PAGE and subsequently transferred onto a polyvinylidene diflouride membrane (Trans Blot Turbo transfer system; Bio-Rad). Membranes were blocked using 5% non-fat milk in a solution of Tris-buffered salt with Tween-20 for 1 h at room temperature. The blocked membrane was incubated with rabbit anti-pIκBα (catalogue no. mAb2859; dilution 1:1500), mouse anti-p65 (catalogue no. mAb6956; dilution 1:1000), mouse anti-β-actin (catalogue no. mAb3700; dilution 1:2000), and corresponding secondary antibody-conjugated horseradish peroxidase. All antibodies were from Cell Signalling Technology Biotechnology Inc., (Massachusetts, USA). The blots were visualized using Western blotting detection system and the protein concentrations in each specimen were normalised to β-actin abundance.

### Statistical analysis

Statistical analyses were performed using GraphPad Prism (GraphPad Software Inc., CA, USA). After checking for normal distribution and equal variance using the Shapiro–Wilk and Levene’s tests, respectively. One-way analysis of variance followed by Tukey’s post hoc test was conducted to examine statistical significance. In addition, to determine the correlation between the cecal microbiota and the transcription level of inflammatory cytokine in bone morrow, correlation analysis was performed using Spearman’s procedure. Data were expressed as mean and standard deviation (SD). *P*-value less than 0.05 was considered significant.

## Results

### RS diet alleviates *E. coli*-induced bone loss primarily by suppressing bone resorption

**Fig. 1:**
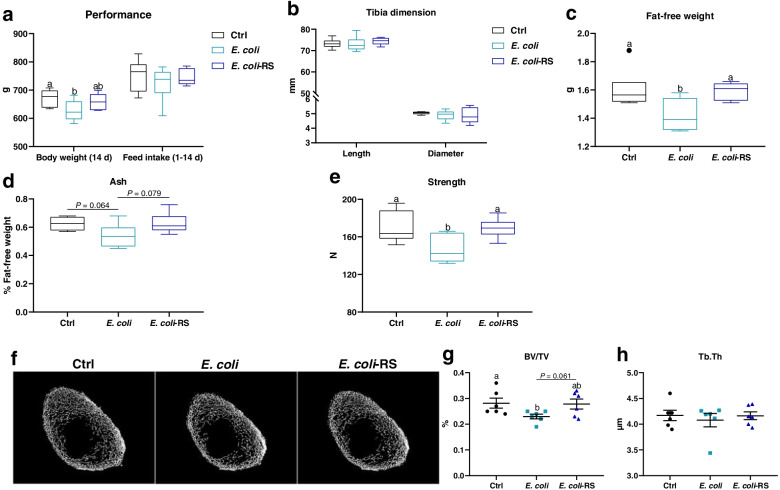
Dietary 12% RPS alleviates *E. coli*-induced bone loss in meat ducks. **a** Performance including body weight at 14 d and feed intake during 1 to 14 d, (**b**) tibia length and diameter, and (**c**) fat-free weight were determined. Bone quality was indicated by (**d**) tibia ash and (**e**) strength. **f** Representative micro CT images and the quantification of **(g**) bone volume/total volume (BV/TV) and (**h**) trabecular thickness (Tb.Th) of the proximal tibia. Data are expressed as mean and standard deviation. ^a,b^Mean values with different letters are significantly different by one-way analysis of variance followed by Tukey’s post hoc test (*P* < 0.05)

To determine the effect of dietary RS on body weight and bone metabolism. Birds challenged *E. coli* were fed basal or 12% RPS diets, and results showed that *E. coli* injection decreased the body weight at 14 d (*P* < 0.05) with comparable feed intake during 1 to 14 d (Fig. 1a). Significant differences in tibia length and diameter were not observed among groups (Fig. 1b). *E. coli-*challenged ducks exhibited lower fat-free weight, ash content (*P* = 0.064), BV/TV, and strength as compared to Ctrl birds (*P* < 0.05). The ducks fed the 12% RPS diet had significantly higher fat-free weigh and increased bone quality compared to birds fed the basal diet under the condition of *E. coli* challenge, as indicated by the increased ash, BV/TV and strength (Fig. 1c-g). However, treatment with *E. coli* or RS did not affect Tb.Th in 14-day-old meat ducks (Fig. 1h). Overall, the decreased bone mass by *E. coli* challenge could be normalized by dietary RS treatment.

**Fig. 2:**
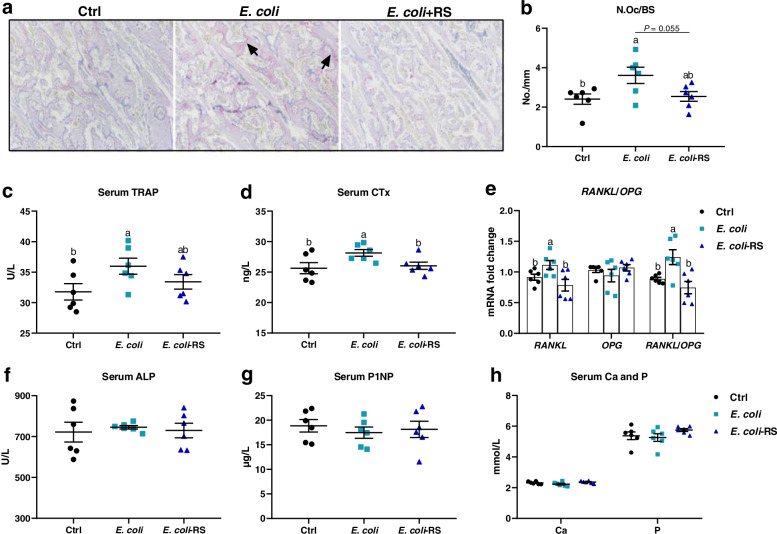
RS diet suppresses osteoclastic bone resorption in meat ducks. **a** Tartrate resistant acid phosphatase (TRAP) staining of tibial sections. Bar = 100 μm. **b** The number of osteoclast (N.Oc/BS) in proximal tibias was determined by histomorphometry. Circulating **(c**) TRAP activity and (**d**) C-terminal cross-linked telopeptide of type I collagen (CTx) concentrations. (**e**) Real-time RT-PCR analysis for mRNA expression of receptor activator for nuclear factor-κB ligand (*RANKL*) and osteoprogerin (*OPG*) in the proximal end, and the ratio of *RANKL*/*OPG* was calculated. Serum bone formation including (**f**) procollagen type I N-terminal propeptide (P1NP) and (**g**) alkaline phosphatase (ALP), as well as (**h**) serum calcium (Ca) and phosphorus (P) were evaluated. Data are expressed as mean and standard deviation. ^a,b^Mean values with different letters are significantly different by one-way analysis of variance followed by Tukey’s post hoc test (*P* < 0.05)

Effects of dietary RS administration on bone resorption were assessed histologically and biochemically. TRAP-positive cells were apparently observed in approximal tibia of ducks injected *E. coli* (Fig. 2a). N.Oc/BS was elevated and subsequently reduced by approximately 34% by the dietary 12% RPS administration (Fig. 2b). Circulating bone resorption markers, TRAP and CTx, were remarkably increased by *E. coli* treatment when compared to Ctrl birds. Supplemented with 12% RPS in *E. coli*-treated birds declined serum bone resorption markers to varying degrees (Fig. 2c, d). Furthermore, the outcome from the mRNA expression of osteoclastogenesis-related factors in bone showed that experimental treatment failed to affect the expression of osteoprogerin (*OPG*) mRNA, whereas dietary 12% RPS supplementation notably decreased the mRNA level of *RANKL*, thereby decreased the *RANKL*/*OPG* ratio induced by *E. coli* challenge (Fig. 2e). In addition, effects of RS treatment on bone formation were also assessed by serum biochemical parameters. No obvious differences in the serum indicator for bone formation including P1NP and ALP were observed among all groups (Fig. 2f, g). About serum Ca and P, our results showed no appreciable changes in these parameters, except tending to increase serum P concentration by dietary 12% RPS administration in *E. coli*-treated birds (Fig. 2h). These data indicate that dietary RS alleviating *E. coli*-induced bone loss may be primarily involved in the suppression of bone resorption.

### RS increases SCFAs production associated with alteration in gut microbiota and enhances intestinal integrity and in *E. coli*-treated birds

**Fig. 3:**
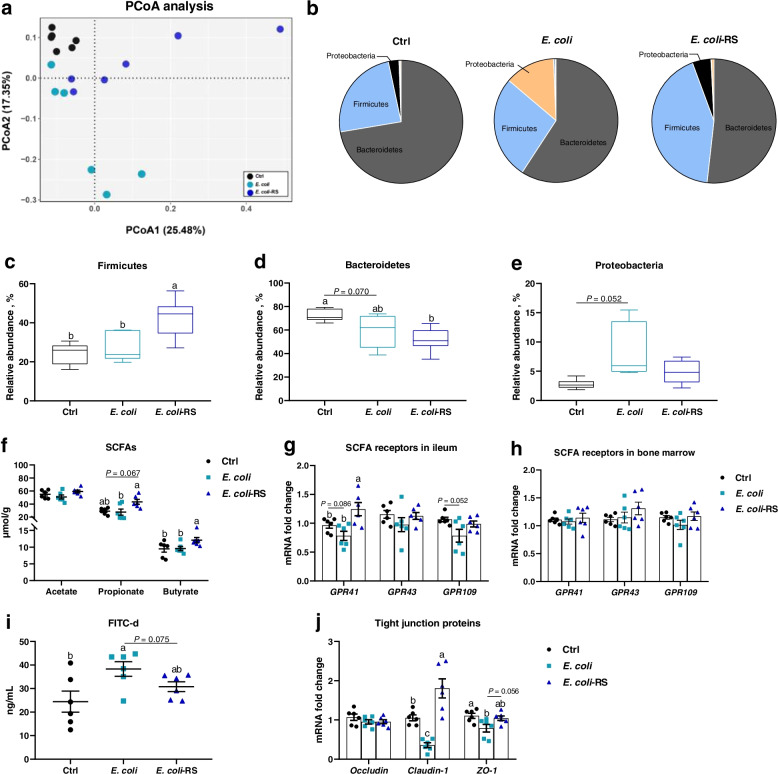
RS changes the microbiota composition and promoting concomitant short-chain fatty acids (SCFAs) production in cecum of meat ducks. **a** Principal coordinate analyses (PCoA) of beta diversity based on Bray–Curtis dissimilarities of bacterial operational taxonomic units. **b** Relative phylum level abundance of gut bacteria. Proportion of (**c**) Firmicutes, (**d**) Bacteroidetes, and (**e**) Proteobacteria. **f** SCFAs production and SCFA receptor gene expression including G protein-coupled receptors 41 (*GPR41*) and *GPR43* in (**g**) ileum and (**h**) bone marrow. Intestinal integrity was also assessed by (**i**) direct measurement using fluorescein isothiocyanate dextran (FITC-d) and (**j**) the mRNA level of tight junction proteins including *occluding*, *caudin-1*, and zona occludens-1 (*ZO-1*). Data are expressed as mean and standard deviation. ^a−c^Mean values with different letters are significantly different by one-way analysis of variance followed by Tukey’s post hoc test (*P* < 0.05)

Using 16 S rRNA sequence analyses to examine gut microbiota changes response to *E. coli* and RPS treatment showed that there was a clear difference in community structure between the Ctrl and *E. coli*-treated ducks (Fig. 3a). Dietary 12% RPS had a strong effect on the gut microbiome yielding a distinctive cluster compared to *E. coli*-challenged birds (Fig. 3a, b). More specifically, the abundance of Firmicutes was dramatically increased in *E. coli*-treated ducks fed 12% RPS diet (Fig. 3c). In contrast, Ctrl ducks had remarkably higher general Bacteroidetes compared with *E. coli*-challenged birds with and without fed RPS diet (Fig. 3d). *E. coli* manipulation increased the proportion of Proteobacteria (which includes *E. coli*) when compared to Ctrl birds (Fig. 3e). Besides, quantifying SCFAs in cecal content was performed, and the data showed that no differences were observed between Ctrl and *E. coli*-treated ducks in terms of SCFAs including acetate, propionate, and butyrate. However, when compared to *E. coli*-injected group, feeding 12% RPS diet modestly increase the concentration of propionate content (*P* = 0.067), and significantly increased the concentration of butyrate (Fig. 3f). Regarding the mRNA expressions of SCFA receptors including G protein-coupled receptor 41 (*GPR41*) and *GPR43* in ileum and bone marrow. Gene expression of SCFA receptors were decreased in the ileum of *E. coli*-treated ducks, which were upregulated by dietary RPS supplementation, especially *GPR41* (Fig. 3g). Whereas there were no obvious differences in bone marrow in terms of the expressions of SCFA receptors mRNA among the Ctrl, *E. coli*, and *E. coli*-RS group (Fig. 3h). These data indicate that RS-caused gut microbiota alteration contribute to the notably elevation in the levels of SCFAs.

Concerned the intestinal integrity, as illustrated in Additional file 1: Table S3, there were no significant differences between Ctrl, *E. coli,* or *E. coli*-RS regarding the length and weight of ileum. However, the 12% RPS diet resulted in lower ileal pH value as compared to *E. coli* ducks. Moreover, direct assessment of permeability using FITC-d suggested that *E. coli* challenge notably increased intestinal permeability, evidenced by higher serum FITC-d concentration and downregulated mRNA level of tight junction proteins (TJPs). Dietary RPS treatment increased the transcription of *claudin-1* and zona occludens-1 (*ZO-1*) and tended to prevent *E. coli*-induced increases in permeability (Fig. 3i, j; *P* = 0.075). Taken together, the RS diet was able to enhance intestinal integrity in *E. coli*-treated meat ducks.

### Alteration in gut microbiota by RS is associated with alleviative inflammatory reaction

**Fig. 4:**
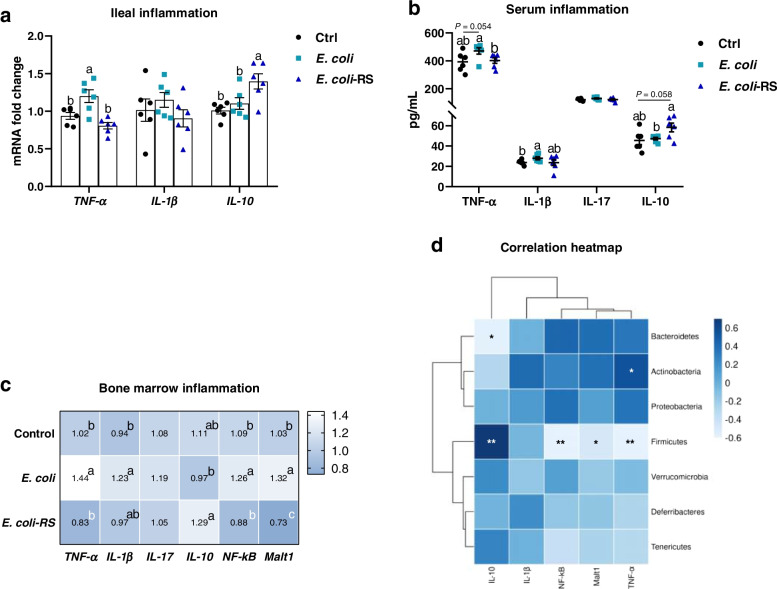
RS is associated with alleviative inflammatory reaction promotes. **a** The transcription of tumour necrosis factor alpha (*TNF-α*), interleukin (*IL*)-*1β* and *IL-10* in ileum. **b** The levels of TNF-α, IL-1β, IL-17, and IL-10 in serum from different groups. **c** The mRNA expression of inflammatory cytokines in bone marrow including *TNF-α*, *IL-1β*, *IL-17*, *IL-10*, nuclear factor-κB (*NF-κB*), and mucosa-associated lymphoid tissue lymphoma translocation protein 1 (*Malt1*). **d** The correlations between the relative abundance of intestinal bacterial genera with the mRNA level of *IL-10*, *IL-1β*, *NF-κB*, *Malt*, and *TNF-α* in bone marrow. Data are expressed as mean and standard deviation.^a−c^Mean values with different letters are significantly different by one-way analysis of variance followed by Tukey’s post hoc test (*P* < 0.05). * and ** denoted significant correlation at *P* < 0.05 and* P* < 0.01, respectively

Given the key role of cytokines in the pathogenesis of *E. coli*-induced bone loss, the impact of RS on pro- and anti-inflammatory cytokines production was also assessed. *TNF-α* was dramatically increased in the *E. coli*-treated birds (*P* < 0.05), however, no significant decrease was detected in the mRNA level of *IL-1β* and *IL-10*. Diet with 12% RPS remarkably depressed *TNF-α* mRNA expression and upregulated the level of anti-inflammatory *IL-10* mRNA (Fig. 4a). Accordingly, the level of proinflammatory factors, including TNF-α and IL-1β, were increased in the serum of *E. coli*-treated birds compared with Ctrl ducks. *E. coli*-injected birds fed 12% RPS diet had a significantly decreased concentration of serum TNF-α. While the anti-inflammatory cytokine IL-10 content showed a trend to increase in RPS ducks compared to *E. coli*-injected birds (*P* = 0.058; Fig. 4b). Reflecting to bone marrow, the higher mRNA levels of *TNF-α*, *IL-1β*, *NF-κB* and *Malt1* in *E. coli*-challenged birds were decreased by the RS diet. In contrast, the mRNA expression of *IL-10* was significantly increased in ducks fed RPS diet relative to *E. coli*-challenged alone birds (Fig. 4c). To better understand the correlation between the abundances of the altered microbes associated with inflammatory cytokines expression in bone marrow, we then performed Spearman analysis and found that the abundances of Firmicutes were negatively correlated with of *TNF-α*, *NF-κB* and *Malt1* mRNA level, and positively related to *IL-10* level, respectively. Bacteroides were negatively correlated with *IL-10* mRNA expression. However, the prevalence of Actinobacteria were positively related to the mRNA level of *TNF-α* in bone marrow (Fig. 4d).

### Suppressing SCFAs production eliminates the favour roles of RS on *E. coli*-induced bone loss through increasing bone resorption

**Fig. 5:**
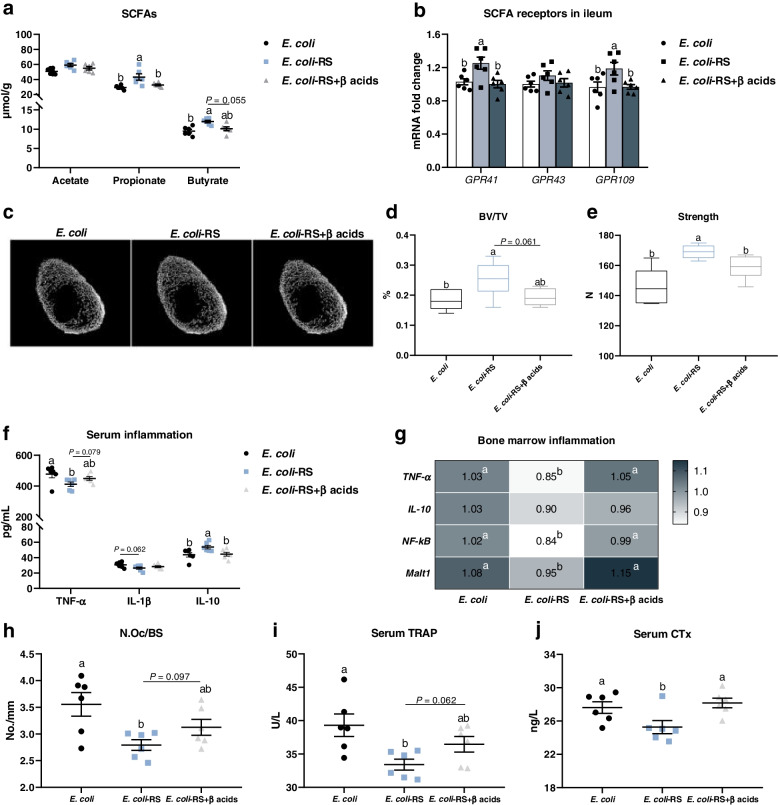
β acid eliminates the improved roles of RS on *E. coli*-induced bone loss via increasing bone resorption. **a** The changes in the levels of short-chain fatty acids (SCFAs) in cecal content and (**b**) in the SCFA receptor gene expression including G protein-coupled receptors 41 (*GPR41*) and *GPR43* in ileum. **c** Representative micro-CT images and the quantification of (**d**) bone volume/total volume (BV/TV) of proximal tibia and (**e**) strength. **f** Serum tumour necrosis factor alpha (TNF-α), interleukin (IL)-1β and IL-10 were detected by ELISA. **g** The inflammatory cytokines *TNF-α*, *IL-10*, nuclear factor-κB (*NF-κB*), and mucosa-associated lymphoid tissue lymphoma translocation protein 1 (*Malt1*) mRNA expression in bone marrow from different groups. **h** The number of osteoclast (N.Oc/BS) in proximal tibias and circulating bone resorption markers, including (**i**) tartrate resistant acid phosphatase (TRAP) and (**j**) C-terminal cross-linked telopeptide of type I collagen (CTx), were also determined. Data are expressed as mean and standard deviation.^a,b^Mean values with different letters are significantly different by one-way analysis of variance followed by Tukey’s post hoc test (*P* < 0.05)

To further confirm whether SCFAs produced by RS-associated gut bacteria are the pivotal factors contributing to alleviation of *E. coli*-induced bone loss. β acid, an inhibitor of bacterial SCFAs production, was used in this study to inhibit SCFAs production. As expected, β acids potently reduced the content of propionate and butyrate in *E. coli*-treated ducks received RPS diets (Fig. 5a). Ileal expression of *GPR41* was decreased in birds given β acids (Fig. 5b). Neither growth performance nor tibia growth were altered by β acids administration, showed by comparable tibial length, diameter, and fat-free weight (Additional file 1: Fig. S1). Dietary RS associated increases in tibia BV/TV and strength were eliminated by β-acids during *E. coli* injection (Fig. 5c-e). Meanwhile, β-acids showed mild increase in serum TNF-α level and obvious decrease in IL-10 concentration (Fig. 5f). Suppressive effects of RPS diet on the mRNA expression of *TNF-α*, *NF-κB*, and *Malt1* in bone marrow were abolished by drinking β-acids manipulation (Fig. 5g). In addition, the inhibited role of RPS on N.Oc/BS and circulating bone resorption markers, including TRAP and CTx, were also eliminated by β-acids addition in different degrees (Fig. 5h-j), whereas the serum bone formation markers ALP and P1NP, as well as Ca content did not changed by experimental treatment (Additional file 1: Fig. S2a-c). Notably, the increased concentration of serum P was significantly reduced in *E. coli*-treated birds receiving RS diet and β-acids compared to *E. coli*-RS ducks (Additional file 1: Fig. S2d). Collectively, these data strongly suggest that SCFAs might be a key regulator for RS attenuating *E. coli*-induced bone loss through suppressing bone resorption mediated by inflammation.

### Dietary RS and Malt1 inhibitor MI-2 attenuate *E. coli*-induced bone loss related to lower bone turnover

**Fig. 6:**
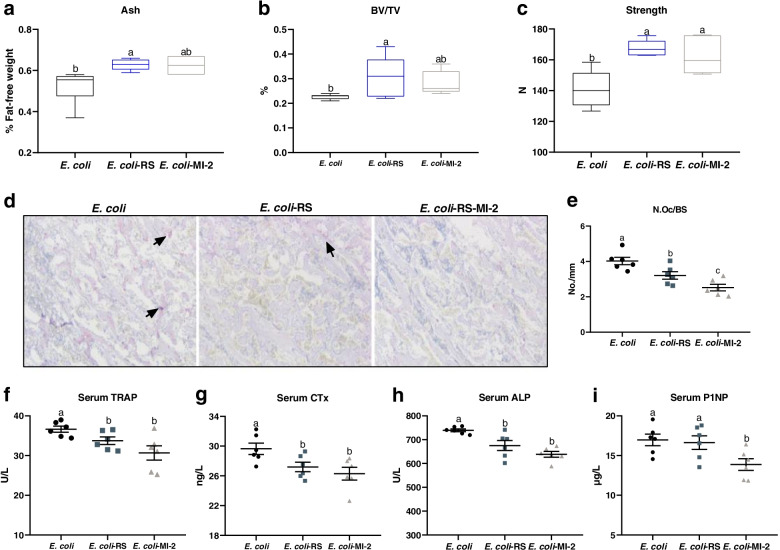
Both dietary RS and lymphoid tissue lymphoma translocation protein 1 (Malt1) inhibitor MI-2 attenuate *E. coli*-induced bone loss related to lower bone turnover. Bone quality was indicated by (**a**) tibia ash, (**b**) bone volume/total volume (BV/TV) of proximal tibia, and (**c**) strength. **d** The proximal tibias was stained using tartrate resistant acid phosphatase (TRAP) staining (Bar = 100 μm), subsequently (**e**) the number of osteoclast (N.Oc/BS) in tibial sections determined by histomorphometry. Circulating bone turnover markers (**f**) TRAP activity, (**g**) C-terminal cross-linked telopeptide of type I collagen (CTx) concentration, (**h**) alkaline phosphatase (ALP), and (**i**) procollagen type I N-terminal propeptide (P1NP) were quantified. Data are expressed as mean and standard deviation.^ a-c^Mean values with different letters are significantly different by one-way analysis of variance followed by Tukey’s post hoc test (*P* < 0.05)

For directly confirming the regulatory role of Malt1 in RS attenuate the reduction of bone induced by *E. coli* in meat ducks, we compared the effects of known Malt1 inhibitors MI-2 and dietary 12% RPS on bone metabolism. As shown in Additional file 1: Fig. S3, with unchanged feed intake during 1 to 14 d, tibia length and diameter, but dietary RS increased the body weight at 14 d and tibia fat-free weight in *E. coli*-challenged ducks. MI-2 administration did not change the performance and tibia growth under the condition of *E. coli* injection. As far as bone mass conacred, dietary 12% RPS-treated *E. coli* ducks had elevated tibia ash, BV/TV, and strength compared to *E. coli* alone birds; MI-2 treated also resulted in numerically increased ash and BV/TV, as well as significantly elevated tibia strength in *E. coli* birds (Fig. 6a-c). Further, there were significant decline of N.Oc/BS in proximal tibia, TRAP and CTx level in serum from *E. coli*-treated ducks receiving dietary 12% RPS similar to the effects of MI-2 (Fig. 6d-g). *E. coli*-challenged birds receiving 12% RPS diet had a significant lower ALP activity but not P1NP level in serum compared to *E. coli* ducks, whereas both serum ALP and P1NP level were significantly reduced by the MI-2 treatment in *E. coli*-treated birds (Fig. 6h, i). These data indicate that both 12% RPS diet and Malt1 inhibitor MI-2 could attenuate *E. coli*-induced bone loss by suppressing osteoclastic bone resorption.

### Dietary RS and MI-2 inhibit Malt1/NF-κB inflammasome activation and modify the splenic T regulatory cell expansion in ducks subjected *E. coli* injection

**Fig. 7:**
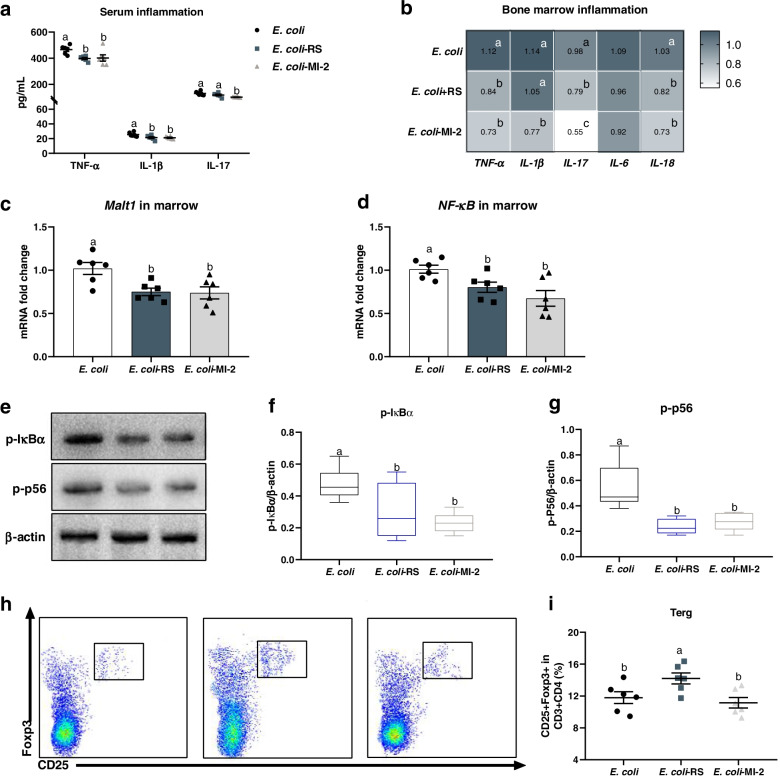
Responses of mucosa-associated lymphoid tissue lymphoma translocation protein 1 (Malt1) mediated nuclear factor-κB (Malt1/NF-κB) inflammasome activation and splenic T regulatory cell (Treg) to dietary RS and MI-2 in ducks subjected *E. coli* injection. **a** The levels of TNF-α, IL-1β, and IL-17, in serum from different groups. **b**-**d** The mRNA expression of inflammatory cytokines in bone marrow including *TNF-α*, *IL-1β*, *IL-17*, *IL-6*, *IL-18*, *Malt1*, and *NF-κB*. **e**–**g** phosphorylation of p65 and IκBα in NF-κB signalling were analysed by Western blotting. **h** Representative flow cytometry plots of Treg cells (identified as CD3^+^CD4^+^CD25^+^Foxp3^+^) and (**i**) quantification of Treg cells in spleen. Data are expressed as mean and standard deviation.^a−c^Mean values with different letters are significantly different by one-way analysis of variance followed by Tukey’s post hoc test (*P* < 0.05)

To further assess the potential mechanism of RS interacted inflammatory response to restore a reduction in *E. coli*-induced bone loss. The cytokine profiles of serum and bone marrow were quantified and showed that both dietary 12% RPS and MI-2 notably decreased the level of TNF-α, IL-1β, and IL-17 in serum (Fig. 7a). Analogous to the effects of MI-2 on inflammatory cytokines mRNA expression, the diet with 12% RPS significantly downregulated the transcription of *TNF-α*, *IL-17*, and *IL-18* in bone marrow (Fig. 7b). Considering the effect of RS on the activation of NF-κB signalling. Both dietary 12% RPS and MI-2 treatment remarkably decreased the mRNA level of *Malt1* and *NF-κB* in bone marrow relative to *E. coli* alone group (Fig. 7c, d). Meanwhile, administration with dietary 12% RPS and MI-2 caused a obvious inactivation in NF-κB signalling, evidenced by lower IκBα and p65 phosphorylation level in the bone marrow from meat ducks given dietary 12% RPS and MI-2 treatment as compared to birds subjected to *E. coli* challenged alone (Fig. 7e-g). Moreover, we quantified the change of Treg cells in spleen, and the RS supplementation caused a significant elevation in splenic Treg cell frequency compared to *E. coli* alone birds. Interestingly, compared with *E. coli* ducks, the proportions of Treg cell in spleen were not altered when birds were administrated with MI-2 (Fig. 7h, i). These results suggest that both 12% RPS and MI-2 reduced *E. coli*-induced activation of Malt1/NF-κB signalling, and Treg cell expansion in RS-fed ducks might be contributor to RS attenuating *E. coli*-induced bone loss.

## Discussion

Dietary changes and supplementation are being considered as useful adjuvant strategies for bone diseases including postmenopausal osteoporosis [32] and arthritis therapy [33]. Ongoing evidence suggest that dietary RS is protective against gastrointestinal disorders, arthritis, and bone loss [28, 30]. Here we report the effects of dietary 12% RPS supplementation as a mediator to regulate “gut-bone” axis on bone metabolism. 12% RPS diet reduced disease pathogenesis of tibia, including slower bone mass and mechanical prosperity induced by *E. coli* challenge, came from suppressing bone resorption mediated by inflammatory cytokines. These effects were closely correlated with the obvious gut microbiome changes associated with dietary RPS in *E. coli*-treated ducks and consequent SCFAs production. As a potential result, SCFAs directly act on intestinal barrier to prevent the onset of *E. coli*-induced inflammatory response via inhibiting Malt1/NF-κB signalling in bone marrow.

The susceptibility to *E. coli* makes meat duck prone to suffer from the intestinal and extraintestinal infections to avian pathogenic *E. coli* leading to inflammatory response [6] and alter cecal microbiome profile [4], which might be potential attributors to bone abnormalities. In the present study, *E. coli* infection caused decreased bone mass and strength accompanying with higher N.Oc/BS, circulating TRAP and CTx level, as well as comparable serum bone formation markers such as ALP and P1NP abundance, suggesting that *E. coli* induced osteoclastic bone resorption and thus impaired tibia quality. In this regard, RANKL binds to RANK that is expressed on the surface of osteoclast to induce osteoclast differentiation, whereas OPG acts as a decoy receptor by blocking the interaction of RANKL with its functional receptor RANK [34]. The higher *RANKL* expression and thereby higher *RANK*/*OPG* ratio in *E. coli* birds probably explained the increased osteoclast number in tibia. In accordance with previous research saying that dietary RS could attenuate the bone loss in ovariectomized mice [26–28], this study also confirmed that dietary 12% RPS supplementation inhibited bone resorption and prevented tibial bone loss induced by *E. coli* injection in meat ducks. Alteration in gut microbiota associated with dietary RPS inclusion might contribute to the beneficial role in alleviating *E. coli*-induced bone loss. Indeed, manipulating gut microbiota via oral antibiotics and production germ-free mice could apparently affect bone quality in mice [10, 11]. Reduced Firmicutes/Bacteroidetes ratio was linked with declined bone loss of mice [35]. In addition, a reduction in the proportion of Firmicutes and an increase in Bacteroidetes were critical correlations with the reduction in disease manifestations in collagen-induced arthritis mice fed RS diet [30]. This contrasts with our research that dietary 12% RPS increased the abundance of Firmicutes with decreased the prevalence of Bacteroidetes in *E. coli*-treated ducks, which was multiply proved by our team studies [24]. These discrepancies might attribute to the experimental animal, diet type, or some unidentified factors. Alternatively, our data implied that dietary RS attenuating *E. coli*-induced bone loss in meat ducks independent of its regulatory effect on the ratio of Firmicutes and Bacteroidetes. Further studies are needed to illustrate these possibilities.

The increased level of SCFAs were likely responsible for the systemic effect of dietary RS on *E. coli*-induced bone loss in meat ducks. Bacteria of the Firmicutes bacteria produce high amounts of butyrate and propionate, whereas Bacteroidetes bacteria produce high levels of acetate and propionate [36]. Consistent with our previous observations, in this study, dietary 12% RPS addition facilized the production of SCFAs in cecal content especially propionate and butyrate [24], and there was a positive correlation between the increased SCFAs and the protective effect RS on collagen-induced arthritis of mice [30]. Reflecting the SCFAs receptors, the gene expression of *GPR41* was significantly increased in the intestine but not in bone marrow, which suggested that SCFAs may act on gut but not directly on bone to interact bone turnover. Published literatures also deemed that a direct effect of SCFAs on bone resorption in vivo is unlikely to interfere the differentiation bone cells [8]. Considered the role of SCFAs in stimulating the secretion of glucagon-like peptide (GLP)-1 and GLP-2 from enteroendocrine L cells to improve intestinal barrier function and/or in exerting anti-inflammation [37, 38], we hypothesized that RS-caused gut microbiota alteration contributes to the notably elevation in the levels of SCFAs, and latter may enhance intestinal integrity to suppress bone resorption mediated by inflammation in bone marrow. Therefore, we evaluated the impact of RPS on intestinal barrier and immune status. As expected, the *E. coli*-induced gut leak, which is in line with previous reports on meat ducks [3], was notably reversed by dietary 12% RPS administration, evidenced by lower serum FITC-d concentration and upregulated mRNA level of *TJPs*. Under the condition of impairing gut integrity, bacteria and their factors probably translocate across the intestinal barrier to induce systemic inflammatory responses [39]. In the current study, using the pro-inflammatory factors (including TNF-α and IL-1β) and anti-inflammatory IL-10 as a marker of the balance between a pro- versus anti- inflammatory state manifested that the increased SCFAs due to RS fermentation exerted anti-inflammatory effect in both intestine and bone marrow. Further, the linking of inflammation and bone metabolism has been well defined [40], e.g., TNF-α, which is secreted along with IL-1 from mononuclear cells, promotes the proliferation of osteoclast indirectly by stimulating RANKL expression and enhancing RANKL binding to osteoclast precursors [41]. Mice with TNF-α induced arthritis were found to have increased circulation of osteoclast precursors which was reversed by anti-TNF-α therapy and correlated with systemically increased TNF-α concentrations [42]. The outcomes from our recent research on broilers indicated that impairment of gut integrity and dysbiosis could induce systemic inflammation to stimulate bone resorption, and consequent result in lower bone quality [7]. For this, in this study, to further confirm whether SCFAs produced by RPS-associated gut bacteria are the pivotal factors contributing to the alleviation of *E. coli*-induced bone loss. The β acid was used and showed that suppressing SCFAs production eliminated the favour roles of RS on *E. coli*-induced bone loss through increasing bone resorption, because β acid was proved to decrease bacterial SCFAs production by gut bacteria without affecting the gut microbial composition per se [43]. Taken together, these data indicate that SCFAs might be a key regulator for dietary 12% RPS attenuating *E. coli*-induced bone loss through suppressing bone resorption mediated by inflammation.

Dependent of the involvement of Malt1, the production of proinflammatory cytokines is governed by NF-κB signalling. Inhibiting Malt1 using MI-2 could prevent the activation of NF-κB signalling thus reduced IL-1β and IL-18 secretion in mice [19]. Of interest, the Malt1 substrates A20 [15] and RelB [16] have previously been associated with osteoclastogenesis. Administration with Mepazine, a Malt1 inhibitor, was found to inhibit the proteolytic activity of Malt1 of mouse BMCs and companying with decreased RANKL-induced formation of osteoclast and the expression of several osteoclast markers, such as TRAP, cathepsin K, and calcitonin [20]. In the present study, our observation that the mitigative effect on *E. coli*-induced bone loss through reducing osteoclastic bone resorption equally well in dietary 12% RPS versus Malt1 inhibitor MI-2 allows us to conclude that Malt1 might be dispensable for dietary RS exerting anti-inflammation and the consequent osteoclastogenesis in meat ducks. Specifically, analogous to MI-2, dietary 12% RPS inclusion decreased the concentration of TNF-α and IL-1β in serum and downregulated the transcription of *TNF-α*, *IL-17*, and *IL-18* in bone marrow owing to the suppression of NF-κB activation, evidenced by lower IκBα and p65 phosphorylation level in the bone marrow from meat ducks given dietary 12% RPS and drinking MI-2. In this context, treatment with MI-2 suppressed bone formation in the current study, showed by decreased serum indicator for bone formation including P1NP and ALP. This suppressive effect on bone formation appears to be an indirect action of MI-2, that is, a consequence of coupling bone resorption to bone formation [44]. Regarding to the relationship between Malt1 and bone mass, as a matter of fact, the patient with Malt1 deficiency had growth inhibition and severe osteoporosis [45]. Deletion of Malt1 in mouse T cells (Malt1^TcellKO^) protects against development of autoimmune arthritis but leads to spontaneous osteoporosis [46]. Malt1 deficient (Malt1^KO^) mice were also noticed to develop an osteoporotic phenotype with increased osteoclastogenesis in vivo [47]. It appears to deem that knockout Malt1 brought about inferior bone quality, independent from a direct role of Malt1 in inflammation mediated osteoclastogenesis, which is completely contrary to our observations. This discrepancy may be accounted by the absence of Treg cells in both the Malt1^KO^ and Malt1^TcellKO^ mice strains, where the Malt1 deficient mice were characterized by the severe loss of Treg cells, the osteoporotic phenotype may rather reflect an indirect effect thereof [46, 48]. Treg cells are well defined to decrease the number of osteoclast both in vitro and in vivo, and lack of Treg cells given rise to bone loss mediated by accelerated osteoclastogenesis in mice [49]. Adoptive transfer experiments of Treg cells in Malt1^KO^ mice could normalize decreased Treg cells account and lower bone density by the abolishment of Malt1^43^. Here, our data showed that MI-2 did not change the amount of Treg cells in spleen, indicated that MI-2 not apparently influenced the Treg cells. The inhibitory effect of MI-2 on osteoclastic bone resorption mainly due to the inactivation in Malt1/NF-κB signalling. In addition, the significantly increased splenic Treg cell frequency in *E. coli*-injected ducks receiving 12% RPS diet implied the Treg cell expansion might be another contributor to RS attenuating *E. coli*-induced bone loss in the current study.

In our study, two new questions need to further address in following research. The first is the addition of SCFAs into drinking water of *E. coli*-treated birds to direct confirm the regulatory role of SCFAs on bone turnover of meat ducks, particularly propionate and butyrate. The second is the assessment of Malt1 on bone mass. There might be differences between genetic and pharmacologic targeting of Malt1 on Treg cell homeostasis and bone phenotype. Previous studies demonstrated evidence that Malt1 deficiency specifically inhibited thymic Treg cell development but having no inhibitory effect on the development of inducible Treg cells in the periphery [48], suggesting that it will be interesting to validate the therapeutic potential of Malt1 targeting on *E. coli*-induced bone loss of meat ducks. Further, specific inhibition of the catalytic activity of Malt1, leaving its scaffold function and thus NF-κB signalling intact, will be also necessary to define the link of Malt1 and bone metabolism in future studies.

## Conclusion

The present study showed that a diet supplemented with 12% RPS alleviates the development of *E. coli*-induced bone loss in meat ducks by changing the gut microbial composition that in turn promoted SCFAs production. Combining the Malt1inhibitor MI-2, our data further indicate that Malt1 might be a master regulator of NF-κB signalling activation and the consequent osteoclastic bone resorption mediated by inflammatory cytokines. Evidence from this study also provided a potential mechanism for “gut-bone” axis and highlighted the importance of dietary fermentable fibbers on bone turnover, which supports the concept that intervention strategies targeting diet adjustment are a valuable approach, and dietary RS intake may be an available adjuvant therapy for osteoporosis in human.

## Supplementary Information

**Additional file 1:** **Table S1.** Composition and calculated nutrient content. **Table S2.** Primers used for real-time PCR quantification of duck target organisms. **Table S3.** The length, weight, and content pH value of ileum response to E. coli and dietary RS treatment. **Fig. S1.** Responses of performance and tibia growth to dietary RS and β acid in ducks subjected E. coli injection. **Fig. S2.** Effect of RS diet and β acid on bone formation, and calcium (Ca) and phosphorus (P) concentration in meat ducks subjected E. coli injection. **Fig. S3.** MI-2 treatment has no apparent effect on performance and tibia growth in ducks subjected E. coli injection.

## Data Availability

All the datasets used and analysed during the current study are included in the manuscript.

## Abbreviations

ALP: Alkaline phosphatase
CTx: C-terminal cross-linked telopeptide of type I collagen
*E. coli*: *Escherichia coli*
IL: Interleukin
Malt1: Mucosa-associated lymphoid tissue lymphoma translocation protein 1
Micro-CT: Micro computed tomography
NF-κB: Nuclear factor κB
P1NP: Procollagen type I N-terminal propeptide
RANKL: Receptor activator of nuclear factor-κ B ligand
RPS: Raw potato starch
SCFAs: Short-chain fatty acids
TNF-α: Tumour necrosis factor alpha
TRAP: Tartrate-resistant acid phosphatase; Treg: Regulatory T cells
