# Supplementary material for: Dietary resistant starch alleviates Escherichia coli-induced bone loss in meat ducks by promoting short-chain fatty acid production and inhibiting Malt1/NF-κB inflammasome activation
Source: J Anim Sci Biotechnol. 2022 Aug 5;13:92. doi: 10.1186/s40104-022-00739-7 (PMC9354418; doi:10.1186/s40104-022-00739-7)
Supplement: Supplementary file 1 — Additional file 1: Table S1. Composition and calculated nutrient content. Table S2. Primers used for real-time PCR quantification of duck target organisms. Table S3. The length, weight, and content pH value of ileum response to E. coli and dietary RS treatment. Fig. S1. Responses of performance and tibia growth to dietary RS and β acid in ducks subjected E. coli injection. Fig. S2. Effect of RS diet and β acid on bone formation, and calcium (Ca) and phosphorus (P) concentration in meat ducks subjected E. coli injection. Fig. S3. MI-2 treatment has no apparent effect on performance and tibia growth in ducks subjected E. coli injection. [file 40104_2022_739_MOESM1_ESM.docx]

**Table S1.** Dietary formulation and composition (as fed basis)

| **Item** | Basal diet | Resistant starch diet |
| --- | --- | --- |
| **Ingredients, %** |  |  |
| Corn | 59.82 | 44.85 |
| Soybean meal | 33.22 | 35.91 |
| Raw potato starch | 0.00 | 12.00 |
| Soybean oil | 0.50 | 1.50 |
| Calcium Carbonate | 1.10 | 1.04 |
| Dicalcium phosphate | 1.75 | 1.82 |
| L-Lysine-HCL | 0.12 | 0.07 |
| DL-Methionine | 0.16 | 0.17 |
| L-Threonine | 0.02 | 0.02 |
| Bentonite | 2.33 | 1.64 |
| Sodium chloride | 0.30 | 0.30 |
| Choline chloride | 0.15 | 0.15 |
| Vitamin premix^1^ | 0.03 | 0.03 |
| Mineral premix^2^ | 0.50 | 0.50 |
| Total | 100.0 | 100.0 |
| **Calculated analysis, %** |  |  |
| Apparent metabolism energy, MJ/kg | 11.72 | 11.73 |
| Crude protein | 19.50 | 19.51 |
| Calcium | 0.90 | 0.89 |
| Non-phytate phosphorus | 0.42 | 0.41 |
| Digestibility Lysine | 1.00 | 0.98 |
| Digestibility Methionine | 0.42 | 0.43 |
| **Nutrient analysis (%)** |  |  |
| Resistant starch | 3.73 | 7.66 |

^1^Provided per kilogram of diet: Cu (CuSO_4_∙5H_2_O), 8 mg; Fe (FeSO_4_∙7H_2_O), 80 mg; Zn (ZnSO_4_∙7H_2_O), 90 mg; Mn (MnSO_4_∙H_2_O), 70 mg; Se (NaSeO_3_), 0.3 mg; I (KI), 0.4 mg.

^2^Provided per kilogram of diet: retinol, 2.06 mg; cholecalciferol, 0.04 mg; vitamin E, 30.01 mg; thiamine, 1 mg; riboflavin, 3.9 mg; pyridoxine, 3.375 mg; vitamin B_12_, 0.01 mg; calcium pantothenate, 8.85 mg; folate, 0.5 mg; biotin, 0.1 mg; niacin, 49.25 mg.

**Table S2.** The primers for quantitative real-time PCR.

| Gene | Gene ID | Primer | Sequence (5′-3′) | Size, bp |
| --- | --- | --- | --- | --- |
| *OPG* | XM_005017709.3 | Reverse | gcctaactggctgaacttgc | 106 |
|  |  | Forward | gaaggtctgctcttgcgaac |  |
| *RANKL* | XM_021276016.1 | Reverse | gccttttgcccatctcatta | 100 |
|  |  | Forward | taagtttgcctggcctttgt |  |
| *ZO-1* | XM_013104939.1 | Reverse | tacgcctgtgaagaatgcag | 86 |
|  |  | Forward | ggagtggtggtgtttgcttt |  |
| *Occludin* | XM_013109403.1 | Reverse | caggatgtggcagaggaatacaa | 160 |
|  |  | Forward | ccttgtcgtagtcgctcaccat |  |
| *Claudin 1* | XM_013108556.1 | Reverse | tcatggtatggcaacagagtgg | 179 |
|  |  | Forward | cgggtgggtggataggaagt |  |
| *Malt1* | XM_027446455.2 | Reverse | ccatggaaaccgtacttgct | 118 |
|  |  | Forward | ttgtgcaggggattggtaat |  |
| *NF-κB* | XM_027455993.1 | Reverse | gagcgttttcaagaggttgc | 123 |
|  |  | Forward | agggatcttctcctgccatt |  |
| *TNF-α* | EU375296.1 | Reverse | agatgggaagggaatgaacc | 51 |
|  |  | Forward | gttggcataggctgtcctgt |  |
| *IL-1β* | DQ393268.1 | Reverse | gcatcaagggctacaagctc | 131 |
|  |  | Forward | caggcggtagaagatgaagc |  |
| *IL-6* | AB191038.1 | Reverse | atctggcaacgacgataagg | 87 |
|  |  | Forward | ttgtgaggagggatttctgg |  |
| *IL-10* | NM_001310368.1 | Reverse | ctgacctcctaccagcgaag | 106 |
|  |  | Forward | gagctgagcagctgaatgc |  |
| *IL-17* | EU366165.1 | Reverse | atgcctgacccaaaaagatg | 145 |
|  |  | Forward | gtggtcctcatcgatcctgt |  |
| IL-18 | XM_027444356.2 | Reverse | ctgatgacgatgagctggaa | 120 |
|  |  | Forward | caaaagctgccatgttcaga |  |
| *GPR41* | KJ523111.1 | Reverse | actgacgtcctcctcctcaa | 160 |
|  |  | Forward | tggtgaggtagatgctggtg |  |
| *GPR43* | KJ523110.1 | Reverse | agcagctgagctttgtcctc | 129 |
|  |  | Forward | gtggaatattaggccgagca |  |
| *β-actin* | NM_001310408.1 | Reverse | ccagccatctttcttgggta | 105 |
|  |  | Forward | gtgttggcgtacaggtcctt |  |
| *GAPDH* | XM_005016745.3 | Reverse | tttttaaccgtggctccttg | 94 |
|  |  | Forward | actgggcatggaagaacatc |  |

*OPG*, osteoprotegerin; *RANKL*, receptor activator of nuclear Factor-κ B ligand; *ZO-1*, zonula occludens; *Malt1*, mucosa-associated lymphoid tissue lymphoma translocation protein 1; *NF-κB*, nuclear factor kappa B; *TNF-α*, tumor necrosis factor alpha; *IL,* interleukin; *GPR*, G protein-coupled receptor; *GAPDH,* glyceraldehyde-3-phosphate dehydrogenase.

**Table S3.** The length, weight, and content pH value of ileum response to *E. coli* and dietary RS treatment

| Items | Ctrl | *E. coli* | *E. coli*-RS |
| --- | --- | --- | --- |
| Length, cm | 64.31 ± 3.06 | 63.31 ± 2.89 | 65.65 ± 1.98 |
| Weight, g | 7.71 ± 0.17 | 7.42 ± 0.28 | 7.65 ± 0.19 |
| pH | 6.48 ± 0.27^a^ | 6.58 ± 0.27^a^ | 6.09 ± 0.49^b^ |

Data are expressed as mean ± standard deviation (SD). ^a,b^Mean values with different letters are significantly different by one-way analysis of variance followed by Tukey’s *post hoc* test.


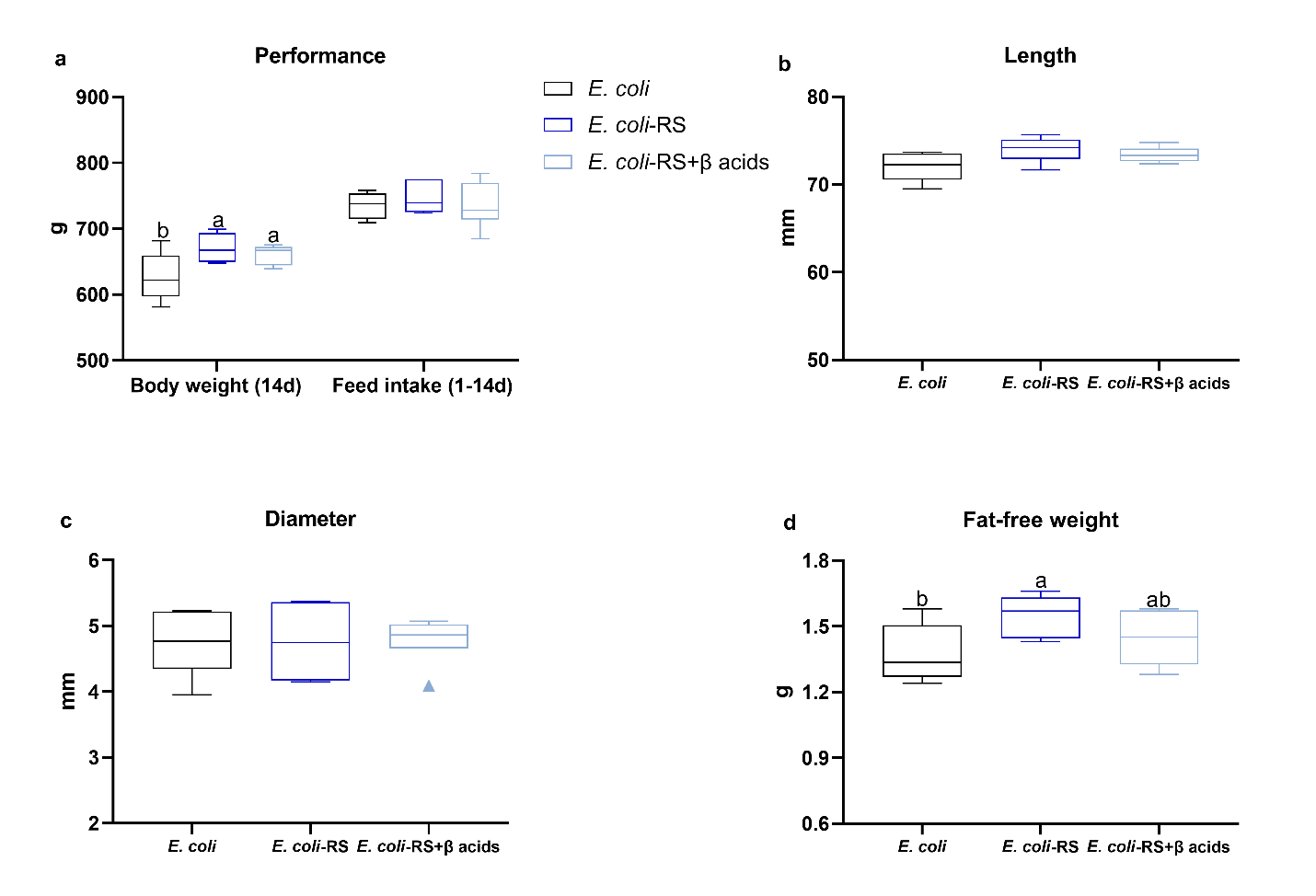


**Fig. S1.** Responses of performance and tibia growth to dietary RS and β acid in ducks subjected *E. coli* injection. (**a**) Performance including body weight at 14 d and feed intake during 1 to 14 d. (**b**) Tibia length, (**c**) diameter, and (**d**) fat-free weight were determined. ^a,b^Mean values with different letters are significantly different by one-way analysis of variance followed by Tukey’s *post hoc* test (*P* < 0.05).


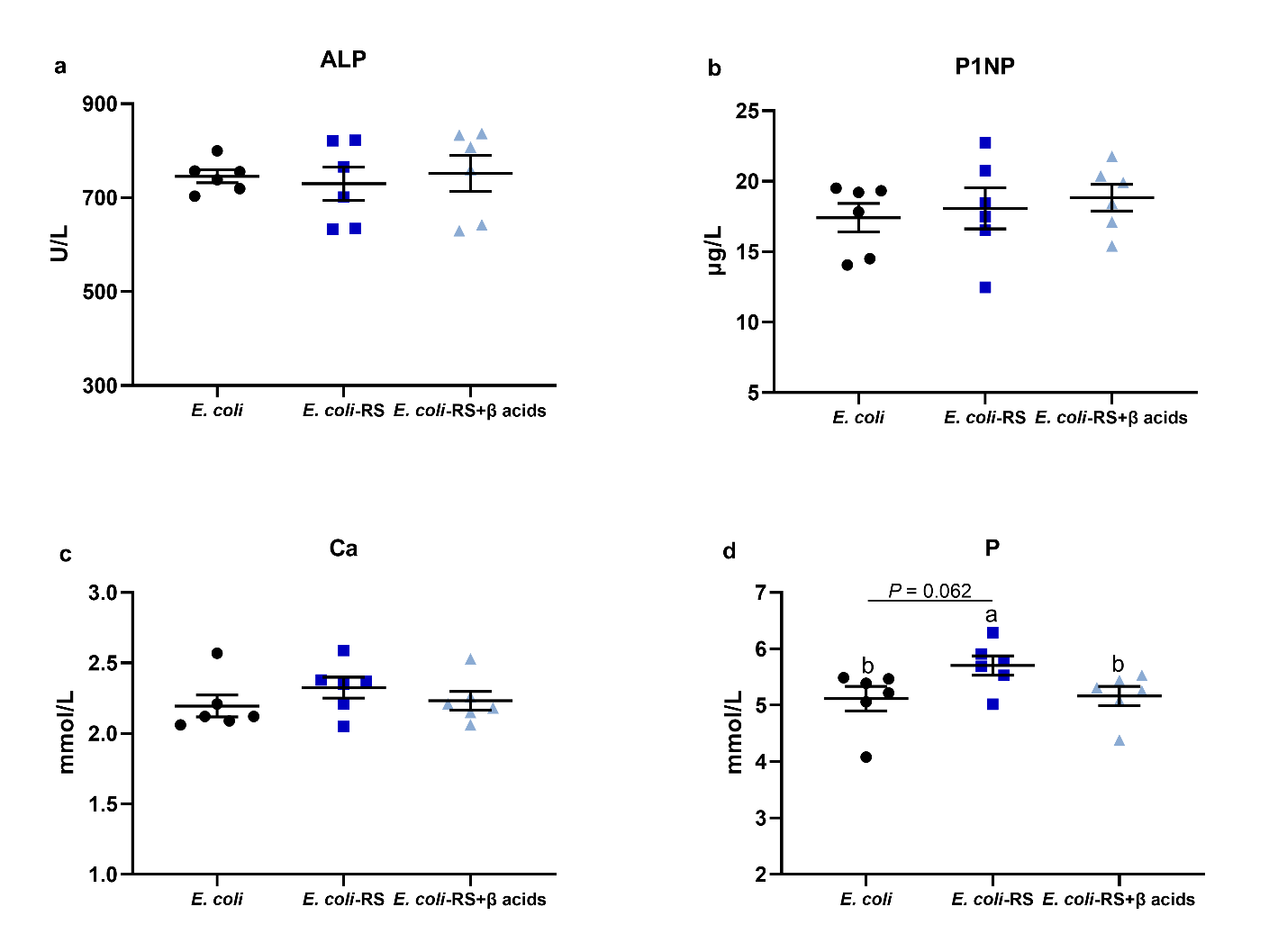


**Fig. S2.** Effect of RS diet and β acid on bone formation, and calcium (Ca) and phosphorus (P) concentration in meat ducks subjected *E. coli* injection. Circulating (**a**) alkaline phosphatase (ALP) and (**b**) procollagen type I N-terminal propeptide (P1NP) level, both reflecting bone formation including, as well as (**c**) Ca and (**d**) P content were evaluated. ^a,b^Mean values with different letters are significantly different by one-way analysis of variance followed by Tukey’s *post hoc* test (*P* < 0.05).

**
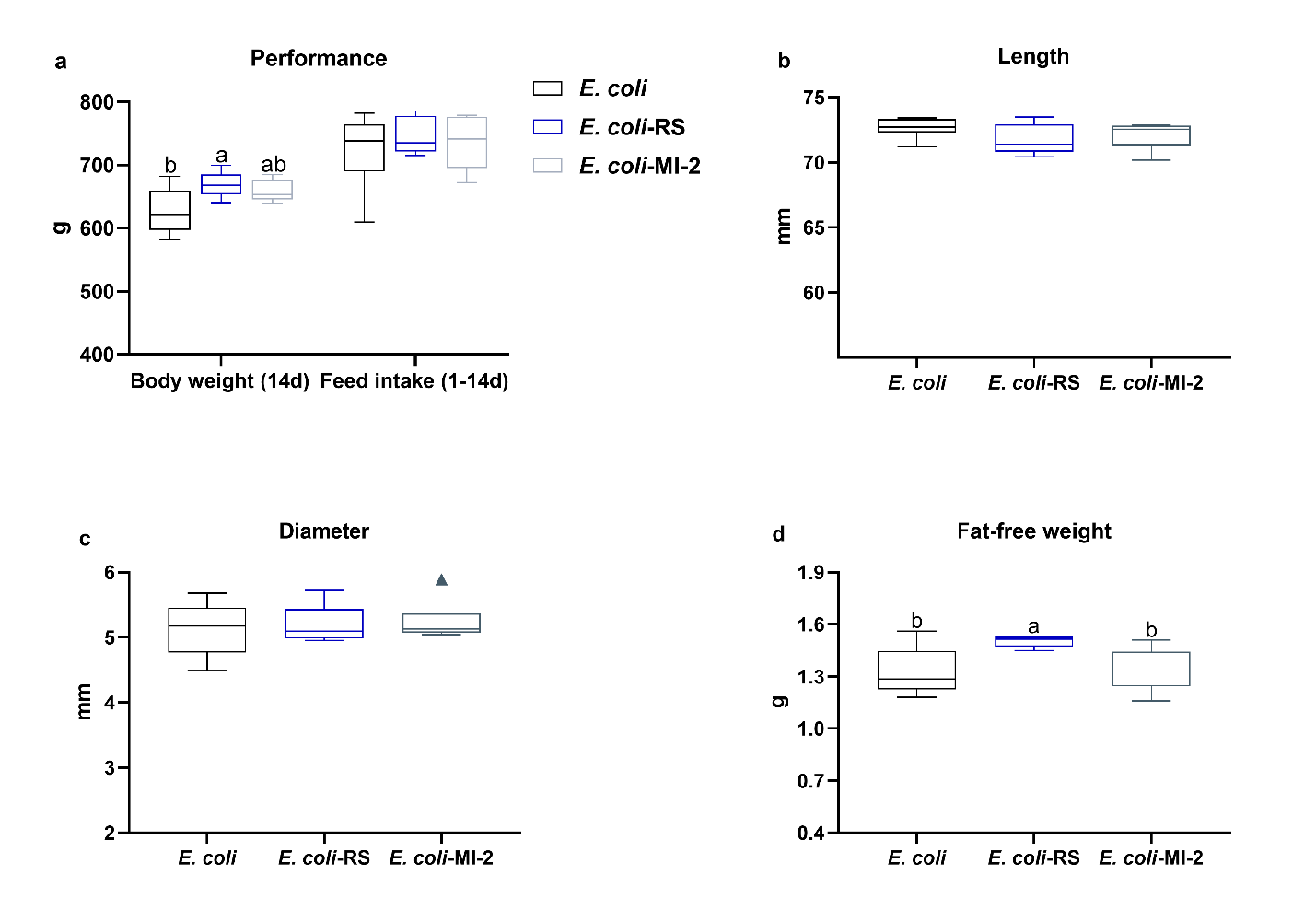
**

**Fig. S3.** MI-2 treatment has no apparent effect on performance and tibia growth in ducks subjected *E. coli* injection. (**a**) Body weight at 14 d and feed intake during 1 to 14 d representing performance were recorded. (**b**) Tibia growth indicted by tibia length (**c**) diameter, and (**d**) fat-free weight were determined. ^a,b^Mean values with different letters are significantly different by one-way analysis of variance followed by Tukey’s *post hoc* test (*P* < 0.05).
